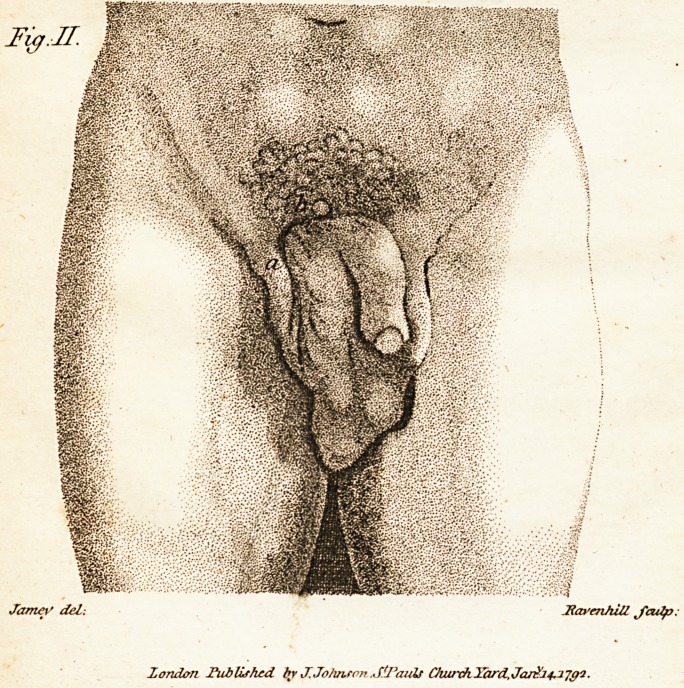# An Account of the Cure of a Preternatural Anus; with Remarks on the History and Treatment of Cases of This Kind

**Published:** 1792

**Authors:** 

**Affiliations:** Surgeon in Chief of the Hotel Dieu at Paris.


XII. *An Account of the Cure of a preternatural Anus; with Remarks on the Hiſtory and Treatment of Caſes of this Kind. By M.* Deſault, *Surgeon in Chief of the Hotel Dieu at Paris.* Vide *Journal de Chirurgie,* Tome I. 8vo. Paris, 1791.

THE ſubject of this intereſting and inſtructive caſe was a ſailor, named Francis Vialtet, who, in May, 1786, while ſerving on board the St. Michael, a ſhip of war, was wounded, in an engagement at ſea, by the burſting of a bomb. The wound extended from two inches above the right abdominal ring to the bottom of the ſcrotum, where the teſticle was laid bare; and at the upper part of the wound a portion of divided inteſtine, of about an inch in length, protruded, but returned into the abdomen again while the ſurgeon of the ſhip was cleanſing the wound.

A month after the accident the patient was brought to the Marine Hoſpital at Breſt, where he remained till the wound was ſufficiently healed to enable him to undertake a long journey on foot to Moulins, the place of his birth. A portion of inteſtine, however, protruded through through the wound, from which fæces were inceffantly difcharged. This protrufion of the inteftine was confiderably increafed by the journey, and his fituation becoming every day more and more diftreffing, he went from one hofpital to another, but without experiencing relief, till, at length, on the 29th of September, 1790, he was admitted into the Hotel Dieu at Paris.

At this time the portion of protruded inteftine had acquired a confiderable bulk. Its fhape was nearly that of a cone nine inches in height, the middle part of which projected a good deal forwards. Its bafis, which was flightly conftricted, came out under a fold of the fkin, a little above the abdominal ring: its apex, which inclined backwards and hung down to the middle of the thighs, terminated in a fmall orifice, through which were difcharged the fæces. From the moment of the accident he had ceafed to void any thing like fæces in the natural way; but it appeared that once in three or four months he had difcharged, by the anus, a fmall quantity of whitifh matter, which feemed to be nothing more than the mucus fupplied by the portion of inteftine next to the anus.

The

The whole ſurface of the tumour was red, and exhibited (more eſpecially at its lower part) an appearance reſembling the valvular one of the inner ſurface of the inteſtines. On the outer ſide of this maſs, another ſmaller tumour, reſembling the other in colour and conſiſtence, was ſeen iſſuing from the ſame abdominal opening. This laſt was of an oval ſhape, but its orifice was ſo conſtricted as to ſuffer only a little moiſture to eſcape. Both theſe tumours had a periſtaltic motion, ſimilar to that of the inteſtines.

The patient, who was tall, and naturally ſtrong and of a good conſtitution, though extremely thin, was obliged, by the violent pain he experienced, at times, in the abdomen, to be conſtantly in a curved poſition, ſo that in walking he ſupported himſelf with a pair of low crutches. An earthen pot, faſtened round his waiſt by means of a cord, and hanging between his thighs, received the extremity of the inteſtine, and the matter that diſtilled into it ſoon acquired an inſupportable fœtor.

M. Default had no difficulty in determining, 1. that the principal tumour was formed by that portion of the inteſtine which correſponded with the ſtomach, and which being invaginated *, ("if," says he, "I may be allowed "so to express myself,") like the finger of a glove turned inside out, presented to the eye its internal surface; 2. that the smaller tumour was formed by the lower part of the intestine invaginated in the same manner; and 3. that the edges of the divided portion adhered to the opening in the parietes of the abdomen, and were united and confounded with them by one common cicatrix.

The long exposure of the parts to the air, the irritation occasioned by the constant discharge of fæces, and by their being rubbed by the patient's cloaths, &c., had so thickened and hardened the membranes, that, our author observes, it would have been hardly prudent to have attempted the reduction of such a mass, if experience had not already taught him how much, in similar circumstances, may be expected from compression. By way of trying its efficacy in this particular case, he compressed the tumour with both his hands for several minutes; and the diminution of bulk he obtained by this method convinced him that a great deal more might be effected, towards the relief of

* *Invaginée.*

the patient, by a well-regulated and long-continued compreffion.

For this purpofe M. Default employed a fimple bandage, with which he covered the whole of the tumour from below upwards, leaving only an opening at the apex fufficient for the paffage of the fæces. The good effect of this method was foon perceptible; for in the evening of the day the bandage was firft applied it became neceffary to tighten it on account of the diminifhed bulk of the tumour; and by the end of the fourth day the inteftine had refumed its natural fize.

M. Default, now thinking it poffible to reduce the inteftine, directed an affiftant to raife the tumour perpendicularly to the opening in the abdomen, and then introducing one of his fingers into the orifice, while with his other hand he prevented the regurgitation of the inteftine, he was enabled gradually to pufh back the whole of it into its natural fituation. The fmaller tumour was then reduced in the fame manner, and with much lefs difficulty.

Notwithftanding this fuccefsful reduction of the inteftine, one great fource of affliction to the patient ftill fubfifted, and this was the continual difcharge of fæces through the wound.

To

To obviate this M. Default introduced into the inteftine a thick tent, made of linen, three inches long, and fupported it by a fuitable bandage. He intended to remove this twice a day, for the purpofe of affording a paffage to the fæces; but it had not been long applied before the patient complained of a great heat in his bowels, and foon after paffed wind by the anus. To thefe fymptoms fucceeded pains like thofe of colic, and a fharp pricking pain in the rectum, attended by an inclination to go to ftool, and he voided, in the natural way, about half a pound of very fluid fæces, fimilar to fuch as are frequently obferved after an indigeftion. In the courfe of the night following he had eight more ftools refembling the firft, all of which were preceded by acute pain about the rectum. The ftools continued to be very frequent during the three following days, but the pain that preceded them became lefs and lefs confiderable, the fæces acquired by degrees a firmer confiftence, and in proportion as this change took place the frequency of the evacuation diminifhed.

The linen tent was retained in the inteftine till the eighth day, when it was removed, and M. Default contented himfelf with covering the external external orifice with a dossil of lint, supported by compresses, over which was applied a broad flat piece of elastic gum. This mode of compression proved sufficient to direct the course of the fæces through their natural channel, and from that time the whole of them continued to be voided by the anus.

The patient now gradually recovered his health and strength, and during the two months he remained in the Hotel Dieu, after this period, his fæces were constantly similar to those of a man in health, and he was perfectly free from complaint. He was repeatedly examined by different surgeons, most of whom had attended to his case from the time of his admission into the hospital, and nothing more was observable in the neighbourhood of the wound in the abdomen than a slight serous exudation which a small part of the lint placed over the orifice had imbibed. Three months after his discharge from the Hotel Dieu, and five from the date of his recovery, he was examined by the surgeon of the hospital at Moulins, to which place he had returned, and was then found to be still in the same good state as when he quitted Paris, although he had lived somewhat intemperately.

Emboldened by the ſucceſsful event of the caſe, the patient began now to think himſelf ſecure from any danger of a relapſe; and accordingly engaged, we are told, in violent exerciſe, and even in feats of activity, in order to make a diſplay of his vigour in the eyes of his townſmen, who had ſeen him only eight months before in a deplorable ſtate. Theſe exertions, as might be expected, produced very alarming effects; for having one day undertaken, for a wager, to lift a caſk of wine upon his knees, his bandage broke, but this did not prevent him from perſevering in his attempt and winning the wager. He walked, it ſeems, for two hours after this with his handkerchief tied round his waiſt; but the inteſtine had begun to make its way out again through the opening which ſtill ſubſiſted in the abdomen, and before he could reach home ſix inches of it had protruded.

He at firſt endeavoured to reduce it himſelf, but without ſucceſs. Different ſurgeons of the town who came to his aſſiſtance likewiſe failed. This was on the 4th of March, 1791, and he immediately determined to attempt to get back to Paris. He began his journey in a waggon, but the pain he experienced from this ſoon obliged him to quit it, and to reſume his former mode mode of travelling, on foot, ſupported by crutches, and with an earthen pot, as before, between his legs to receive the fæces.

In this condition he reached the Hotel Dieu at Paris, and came again under the care of M. Deſault, on the 31ſt of March. The hardneſs of the tumour at this time, we are told, was as great, though its bulk was not ſo conſiderable, as when he was admitted the firſt time. After bleeding him, recourſe was had, as before, to a gradual compreſſion of the inteſtine by means of a bandage; but ſix days elapſed before the inteſtine was in a ſtate that would allow M. Deſault to attempt its reduction. It was then effected, however, it ſeems, without much difficulty; and compreſſes of lint, linen, and elaſtic gum, properly ſupported, were applied as before. The reduction of the inteſtine was immediately followed by nauſea and vomiting, which ſubſided in about two hours, when the patient had a copious evacuation by ſtool, of very liquid fæces, which was preceded, as formerly, by pains in the bowels, particularly about the rectum. During the following night and day he had a diarrhœa, which leſſened on the ſecond day, and the fæces then began to reſume their natural conſiſtence.

On the 9th of April, the day the account of the cafe was written, the fæces were continuing to pafs in the natural way, without the leaft difcharge from the wound, and the patient, M. Default affures us, was in every refpect as well as before his relapfe, fo that he propofed to fend him out of the hofpital again in a few days.

To illuftrate his account of the cafe M. Default has added two figures of the ftate of the parts before and after the cure, which our readers will find accurately copied in the annexed engraving, the references to which are as follow:

In fig. 1. the letters *a*, *b*, *c*, *d*, *e*, mark the principal tumour formed by the part of the inteftine next to the ftomach, turned outwards upon itfelf like the finger of a glove.

*a*, *e*, the bafis fupporting the broadeft part of the tumour, and iffuing from under a fold of the fkin.

*b*, rugofities formed by the villous membrane of the inteftine.

*c*, the

Fig: I.

Fig. II.

Jamey del: Ravenhill ſculp:

London Published by J. Johnson, St. Pauls Church Yard, Jan. 14. 1792.

*c*, the apex of the tumour, having a ſmall opening poſteriorly through which the fæces iſſued.

*e*, *f*, the penis puſhed towards the left by the tumour.

*g*, the ſmaller tumour formed by the part of the inteſtine next to the anus.

Fig. II. repreſents the ſtate of the parts, in December, 1790, after the cure of the patient.

*a*, *b*, a fold of the ſkin forming a ſort of valve before the opening in the abdomen, which remained fiſtulous.

At the time the preceding caſe occurred in the Hotel Dieu M. Deſault had under his care there another man with a preternatural anus that had been formed eleven years before, in conſequence of a ſcrotal hernia, the ſtrangulation of which had terminated in gangrene. In this caſe a portion of the inteſtine next the ſtomach had protruded with its villous membrane outwards, as in the caſe of the ſailor, and formed a tumour of three inches in length; but the other portion of the inteſtine did not appear. This patient, M. Deſault obſerves, was lean and feeble, although he devoured a prodigious quantity of food, but it ſeems he conſtantly voided it in an imperfectly digeſted ſtate, and to this circumſtance our author thinks it was owing that he preferred food of difficult digeſtion, particularly ſallad.

This man, encouraged by the ſucceſsful termination of the ſailor's caſe, entreated M. Deſault to undertake the treatment of his, and it was accordingly attempted, though with little expectation of ſucceſs, as a portion of the inteſtine contiguous to that which protruded, and which had formerly fallen down into the ſcrotum, had contracted ſuch an adheſion to the ſurrounding parts, that it was found impracticable to make any preſſure on the opening in the inteſtine without at the ſame time ſubjecting this adhering portion to compreſſion. M. Deſault ſucceeded, however, in the reduction of the protruded portion of inteſtine, and its opening was cloſed with a linen tent, ſupported by means of a truſs. Eighteen hours after this the patient experienced ſlight colic pains in his bowels, which alarmed him ſo much, that he removed the tent, and gave up all hopes of cure.

This attempt, however, our author obſerves, ſlight as it was, ſeemed to have produced a ſenſible effect. The patient, who before had been accuſtomed to void, about once in four months, a whitiſh mucus by the anus, had been obliged that day to go twice to the cloſe ſtool, and had voided each time as much of this mucus as he uſed to do when the intervals between this kind of ſtools were very long. The ſame thing, we are told, happened during the eight following days. The intervals afterwards became gradually longer, and at the time (April, 1791) the account was written he had paſſed a whole month without any ſuch mucous diſcharge.

From this caſe, M. Default candidly acknowledges, no concluſion can be drawn; but in a diſeaſe ſo little underſtood, as the one in queſtion, every well-authenticated fact, he obſerves, becomes of importance.

At the time he was writing this paper he had, he tells us, two other caſes of preternatural anus under his care, of different kinds, but both of them very complicated, and likely to throw conſiderable light on the diſeaſe in queſtion, and on the manner of treating it. One of theſe patients, he adds, already voided fæces by the rectum, and although there was ſtill a flight difcharge from the opening in the abdomen, every thing feemed to announce an approaching cure.

This difeafe of the preternatural or (as it is more commonly, though perhaps improperly, called) *artificial* anus affords, in the opinion of our author, a new field of obfervation to the practitioners of furgery. The writings of the ancients, he remarks, furnifh us with very few inftances of fuch an affection. They occur, he acknowledges, more frequently in the writings of the moderns, but in general, he obferves, we find even in thefe only the occafional caufe of the difeafe, and the external appearance of the parts mentioned, without any defcription of the ftate of the inteftine, a circumftance on which he feems to lay the greateft ftrefs. One of the moft frequent occurrences in this complaint, the protrufion of the inteftine out of the belly, feems, he obferves, even to have efcaped the notice of every writer from the time of Hippocrates, who has defcribed it *, to that of Fabricius Hildanus, who, at the beginning of

* Epidem. lib. vii.

the laſt century, related an example of it as a thing unknown and altogether extraordinary*.

Although we find the writers ſince the time of Fabricius frequently ſpeaking of tumours formed externally by the inteſtine, yet it is only within theſe few years, our author contends, that the ſtate of the parts conſtituting ſuch tumours has been aſcertained. M. Robin, he obſerves, found the cæcum and part of the colon invaginated in the rectum, in a hernia of the latter inteſtine that proved fatal. This fact, which we find related by M. Hevin in the fourth volume of the *Memoires de l'Academie de Chirurgie* †, and another ſimilar one deſcribed by M. Le Blanc, would, he thinks, have been ſufficient to point out to us the ſtate of the inteſtine in theſe caſes, even if M. Le Cat had not had occaſion to diſſect the dead body of a woman who had a preternatural anus, and to convince himſelf of the invagination of the inteſtine, which in that caſe had protruded itſelf externally ‡.

* Centur. 1. obſ. 74.

† Edition in 4to.

‡ Philoſophical Tranſactions, No. 480, p. 716.

The effects enumerated by our author as attendant on a complaint of this kind, even in its simplest form, are, the extreme uncleanliness occasioned by the incessant discharge of fæces through the preternatural opening; the excoriation of the surrounding parts; the frequent return of griping pains to which the patient in these cases is subject; the difficulty with which the fæces are sometimes evacuated on account of the narrowness of the opening; and lastly, the reduced state of the patient's strength, in consequence of imperfect digestion, and which sometimes terminates in a fatal marasmus, as in the cases related by M. M. Hoin and Le Blanc *, and as our author himself had occasion to see lately in the Hotel Dieu at Paris.

These inconveniences, he observes, have induced surgeons to attempt different modes of relief in such cases. Receptacles made of silver, of tin, and, what is still better, of elastic gum, applied to the opening in the abdomen, and supported by a suitable bandage, have, in different cases, rendered the condition of the patient less offensive to himself and those about

* Essai sur les Hernies, 1768.

him, him, by collecting the fæces, and thus in some measure lessening the fœtor; and in one case M. Moscati is said to have been able to retain in the opening in the belly a leaden canula through which the fæces were conveyed into a receptacle of tin *.

M. Sabatier, it seems, proposes †, in these cases, to keep, by means of a tent of sufficient bulk, the opening in the intestine large enough to allow an easy passage to the fæces. On the other hand, Professor Richter advises us (with a view to render the digestion more complete) to check the discharge of fæces by means of a piece of sponge applied to the external opening, and supported by an elastic bandage ‡. This method, our author observes, though very ingenious, has been condemned by M. Leffler, who has seen it followed by pains and constipation of the bowels, and by inflammation and excoriation of the skin.

Some few practitioners, M. Default remarks, not satisfied with these palliative methods, have been induced to attempt the radical cure which

* Mem. de l'Acad. de Chir. Tom. V. p. 596.

† Ibid. p. 594.

‡ Traité des Hernies, traduit par Rougemont, chap. xxviii.

nature nature herself, in these cases, seems to point out. Numerous instances, he observes, prove that the fæces have oftentimes resumed their natural course after having been discharged, even for many months, from the wound in the abdomen that has remained after an operation for a hernia. He particularly refers to a case described by the late M. Petit, in which the two ends of the intestine hanging out of the ring, after the separation of the gangrenous parts, became covered with fleshy granulations, and were gradually confounded with the surface of the wound by a common cicatrix, the fæces in the mean time resuming their course by the anus, without any assistance from art *. M. Default has found an account of another fact of the same kind related by Acrel in his Cases of Surgery †; and he refers for similar instances to the writings of Le Dran, (*Obs. de Chirurg.*); Pott, (*Treatise on Ruptures*); Richard, (*Obs. de Med.*); the *Journal de Medecine*, (Tomes XXXII. & XXXVII.); the Transactions of the Society at Harlem, (Tom. I.);

* Malad. Chir. Tom. II. p. 407.

† Chirurg. Handelser. p. 174.

and and the Eſſays publiſhed by a Chirurgical Society at Copenhagen.

The reſources of nature in ſo great a number of inſtances could not fail, our author remarks, to excite the efforts of art; and he thinks it probable that the want of ſucceſs in the firſt attempts depended principally on the defective method adopted by practitioners, who ſeem not to have ſufficiently conſidered the nature of the diſeaſe. Some, he obſerves, not properly attending to the *invagination* of the inteſtine, (we uſe his own expreſſion) have propoſed to bring together its divided portions, while out of the abdomen, according to Ramdhor's method, and to reduce them when ſufficiently united *. Others have conſidered a ſtrict regimen as the moſt likely means of healing the opening in the abdomen, by diminiſhing the diſcharge of fæces. We find our author, however, ſpeaking of both theſe methods as matters of mere ſpeculation which have never been reduced to practice.

M. Default has found, in the letter addreſſed by M. Bruns to M. Henkel, a caſe of preternatural anus, the edges of which, after having

* Richter, chap. xxviii. p. 162.

been been previoufly excoriated by the application of lapis infernalis, were kept together by means of a future. In this way, it feems, a reunion was effected, but it was not of long duration, as the wound burft open again before many days had elapfed.

M. Le Cat alfo, our author obferves, thought of attempting the cure of a woman who laboured under a complaint of this kind, by excoriating the edges of the wound and bringing them together by means of a future, after having previoufly dilated, by means of a canula, the portion of inteftine correfponding with the anus. But this portion, it feems, had acquired fo confiderable a bulk, that it refifted violent efforts to reduce it, and the patient was fo much alarmed by the failure of thefe endeavours to relieve her, that fhe refufed to fubmit to any farther trials *.

Thefe unfuccefsful cafes, M. Default obferves, deterred furgeons from making any new attempts in cafes of this kind, and they began, at length, to be perfuaded that the cure of thefe complaints was impracticable, or at leaft

* Philofophical Tranfactions, in the place already quoted.

attended with great danger to the life of the patient.

Several practitioners, he remarks, have gone ſo far as to conſider even the reduction of the inteſtine as dangerous; and all of them have ſuppoſed it to be impracticable, when the tumour has been of long ſtanding and its bulk conſiderable. It has even, he adds, been aſſerted, that the portion of inteſtine next to the rectum oftentimes cloſes, and that its cavity becomes obliterated. Our author has found even Profeſſor Richter falling into this miſtake, although the invagination, which he ſuppoſes to exiſt, ſeems to be a deciſive proof of the exiſtence of a cavity. This pretended obliteration, however, M. Deſault aſſures us, is ſo far from being ſupported by facts, that all thoſe he is acquainted with ſeem to prove that it cannot poſſibly take place. M. Le Cat, he obſerves, diſcovered no appearance of any ſuch obliteration in the body he opened twelve years after the fæces had ceaſed to paſs through the rectum; and in the patient whoſe caſe has been already mentioned as having died in the Hotel Dieu of maraſmus in January laſt, the inferior portion of the inteſtinal canal was found, we are told, entire, though a little contracted.

In

In that cafe a confiderable portion of the ileum had been deftroyed by gangrene, and nothing had paffed through the rectum for more than two years before the patient's death. It is moreover remarked by our author, that all the patients, of whofe cafes we have any accurate account, have voided from time to time, by the anus, the mucus of the inteftine; and this fact alone he confiders as an evident proof that the cavity in fuch cafes is not obliterated.

Some authors, he obferves, from not having properly attended to the ftate of the inteftine, feem to think that it protrudes out of the belly in its ordinary ftate, and that it is not its extremity that adheres to the fkin; and hence their fears left the fæces, or the mucus of the portion of inteftine next to the rectum, fhould find their way into the cavity of the abdomen. On this fubject he refers to Profeffor Richter's work * already quoted.

The thickening of the membranes of the inteftine he confiders as a more ferious objection. This, he obferves, has always been looked upon as an invincible obftacle to the reduction; but the cafe juft now defcribed proves, he thinks,

* Chap. xxix.

fufficiently, fufficiently, the poffibility of getting back the inteftine into the cavity of the abdomen ; and he is of opinion that even without any fuch inftance we might have been led to attempt it from obferving the effects of a fimilar mode of treatment in cafes of *prolapfus ani* of long ftanding, and which, though feemingly irreducible, on account of their bulk, have been found to give way to a graduated compreffion.

The number and extent of the adhefions, which have infpired fome practitioners with fo much fear, ought not, our author contends, to prevent the reduction. For fuppofing them to exift, and to be even more dangerous than thofe which inflammations of the lower belly almoft always produce, he does not fee what advantage could be expected to refult from leaving the invaginated portion of the inteftine out of the abdomen. Befides, fuch a practice, he obferves, may be productive of the moft dangerous and even fatal effects. In proof of this he refers to M. M. Puy *, Hoin, and Le Blanc †, who, in different cafes, have feen the fwelling and inflammation of the protruded gut fo confiderable

* Mem. de l'Acad. de Chir. Tom. V.

† Operat. de Le Blanc, Tom. II. p. 445.

as to occaſion death; to M. Lange *, who, in another inſtance, found the inteſtine ſo inflamed and tumefied, that it became neceſſary to remove the ſtricture by means of an inciſion in the abdomen; and laſtly, to the caſe of the invalid, mentioned by M. Sabatier in his memoir on this ſubject †, whoſe life was endangered from a ſimilar cauſe.

He conſiders it, therefore, as demonſtrated, (and he ſpeaks of this as the principal point for which he contends) that, in the caſe of a preternatural anus, ſound practice requires the inteſtine to be replaced within the belly, and that ſuch a reduction is always poſſible, whatever may be the ſize of the tumour, and however long it may have exiſted.

There remains, therefore, he thinks, only to be conſidered the moſt convenient and eaſy mode of retaining the inteſtine, and preventing it from again protruding. The ivory peſſary which has been propoſed for this purpoſe, he obſerves, by no means fulfils this indication, as the inteſtine is ſtill liable to eſcape through the aperture in the inſtrument, which would thus

* Schmucker, Vermiſchte Chirurg. Schriften. Tom. II.

† Mem. de l'Acad. de Chirurgie, Tom. V.

become become a new means of ſtrangulation. Beſides, he adds, the preſſure of ſo hard a ſubſtance muſt neceſſarily affect the ſurrounding parts, and ſoon render it inſupportable, at leaſt if the preſſure made by it is ſufficient to fulfil the purpoſe it is introduced for, (but on what good grounds our author thinks it would be difficult to ſay) that of ſupporting the edges of the opening in the abdomen.

The ſoft cuſhion recommended by M. Sabatier, and the ſponge employed in theſe caſes by Profeſſor Richter, are not liable, M. Deſault obſerves, to the ſame diſadvantages; but they both of them, he adds, are attended with the inconvenience, remarked by M. Leffler, of imbibing a portion of the thin and acrid matter that paſſes through them, and which muſt unavoidably excoriate the adjacent parts.

Our author next proceeds to examine the apparatus he himſelf adopted, of the tent made of linen, with the addition of a layer of lint, and other compreſſes, properly ſupported by a bandage of ſufficient tightneſs. Such an apparatus, he obſerves, while it completely prevents the protruſion of the inteſtine, will conſtantly keep up a ſufficient dilatation, by retaining the fæces in the interval of the dreſſings, and occafion them to remain long enough in the inteſ-tinal canal to afford nouriſhment to the patient. If a little fluid ſhould chance to eſcape, it will, he remarks, be abſorbed by the lint, and of courſe will occaſion no irritation on the ſkin. The patient, he adds, will ſoon be accuſtomed to the ſort of reſtraint which this apparatus will at firſt neceſſarily occaſion; and the ſlight colics which muſt be expected to follow the early uſe of it will, he aſſures us, ceaſe in a very few days.

M. Default acknowledges that his views did not extend beyond theſe palliative effects when he firſt adopted this mode of treatment; but the unexpected ſucceſs he experienced from it in the caſe which forms the principal ſubject of the paper before us carried his ideas ſtill farther, by ſhowing him the poſſibility of curing, at leaſt ſometimes, a diſeaſe hitherto conſidered as irremediable, and at the ſame time proving to him the little inconvenience, the advantage even, which might be expected to reſult, in every caſe of this kind, from undertaking the cure by the ſafe and ſimple means he has deſcribed, and varying them according to circumſtances.

In the preternatural anus, whether it be the consequence of a penetrating wound of the abdomen, or of a hernia that has terminated in gangrene, there can be only two ſtates of the inteſtine, he obſerves, eſſentially different from each other. One of theſe, and the moſt common, is when only a part of the circumference of the gut has been injured; the other is when the inteſtine has been completely divided. In each of theſe caſes, our author remarks, an adheſive inflammation unites the edges of the ſection of this canal with the edges of the wound of the integuments and other ſurrounding parts, and from that period the parietes of the abdomen, if they remain entire, will prove a ſupplement to the portion of the canal which has been deſtroyed, and the fæces will continue to be voided by the anus, unleſs the portions of the inteſtine ſhould, by their mode of adheſion, form an angle ſufficiently acute to ſtop them in their progreſs.

The wound of the abdomen, from its affording an eaſier and ſhorter iſſue to the fæces than if they had to paſs through all the circumvolutions of the inteſtines, and the morbid ſtate of that canal, are, therefore, in our author's opinion, to be conſidered as the efficient cauſes of the preternatural anus, or, in other words, of the difcharge of the fæces through the abdominal opening. But to thefe primary caufes, he obferves, is foon added another, which, though fecondary, is not lefs powerful in its effects. This is the contraction which takes place in that part of the inteftine correfponding with the rectum.

But are thefe caufes, our author afks, fo powerful that the furgeon fhould be deterred from attempting to overcome them? The firft of the three, that is to fay, the opening in the abdomen, cannot, he obferves, be an invincible obftacle, becaufe it often happens, in hernias attended with gangrene, that the fæces refume their natural courfe after having paffed for fome time through the wound; and he is of opinion that, in the prefent improved ftate of furgery, an accident of this kind is not fo often followed by a preternatural anus as formerly.

A tent of fufficient bulk, to act as a plug, may therefore, he obferves, fupply the want of continuity in the parietes of the abdomen; but this, he adds, is not all: for the two portions of the inteftine often form an angle at the part where the feparation has taken place; and this this angle, as M. Morand formerly remarked*, affords more or lefs refiftance to the paffage of the fæces in proportion as it is more or lefs acute, and the endeavours of the furgeon to remove this obftacle, and direct the fæces again into their natural channel, will, our author contends, be fuccefsful only in proportion as he is able to enlarge the angle formed by the fegments of the inteftine, by feparating them from each other. Long tents, or plugs, made of lint or linen, introduced and fixed in the two ends of the inteftine, will, in M. Default's opinion, be fufficient to fulfil this indication, by bringing together gradually the divided portions of inteftine in a ftraight line. By this method alfo, he thinks, the upper end of the portion of inteftine correfponding with the rectum will be dilated, and thus the fæcal matter will gradually make its way to the natural anus.

M. Default, towards the conclufion of his paper, indulges himfelf with a hope that the method of treatment he has defcribed may be the means, perhaps, of reftoring to fociety a great number of thofe unfortunate perfons who

* Mem. de l'Acad. des Sciences, 1735, p. 249.

labour under the complaint in queſtion. Even ſuppoſing that this method can never be completely ſucceſsful, (a ſuppoſition, however, which he thinks is ſufficiently contradicted by the caſe he has deſcribed of the ſailor) ſtill, he contends, it can be productive of no inconvenience, and the patients who have recourſe to it will, at any rate, he obſerves, derive from it the advantage of being able to retain, at will, the alimentary matter, ſo as to be no longer in danger of dying of inanition, and will be ſecure from the conſequences of a ſtrangulation, which are always alarming, and ſometimes fatal.

## Figures and Tables

**Fig: I. f1:**
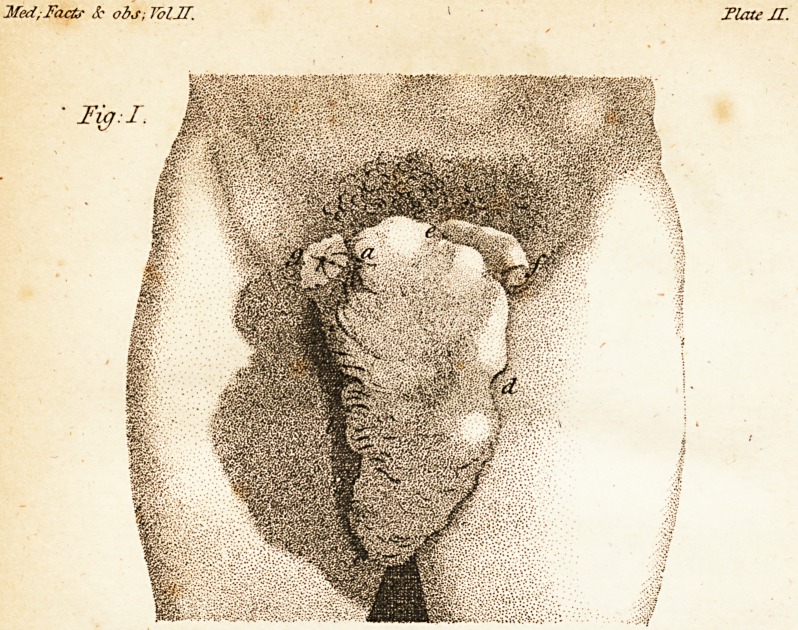


**Fig: II. f2:**